# A narrative review of hydrogen therapy for COPD: aging-related insights

**DOI:** 10.1515/med-2026-1438

**Published:** 2026-05-20

**Authors:** Ya-Ting Chuang, Chun Tseng, Tao-An Chen

**Affiliations:** Surgical Intensive Care Unit, Department of Nursing, Show Chwan Memorial Hospital, Changhua City, Changhua County, Taiwan; Division of General Surgery, Department of Surgery, Show Chwan Memorial Hospital, Changhua City, Changhua County, Taiwan; Doctoral Program in Industrial and Smart Technology, National Chung Hsing University, Taichung City, Taiwan; Division of Respiratory Therapy, Department of Chest Medicine, Show Chwan Memorial Hospital, Changhua City, Changhua County, Taiwan; Institute of Emergency and Critical Care Medicine, National Yang Ming Chiao Tung University, Taipei City, Taiwan

**Keywords:** molecular hydrogen, chronic obstructive pulmonary disease, aging, oxidative stress, inflammation

## Abstract

**Objectives:**

Molecular hydrogen (H_2_) exhibits selective antioxidant, anti-inflammatory, and anti-senescence properties that may provide therapeutic benefits for chronic obstructive pulmonary disease (COPD), particularly in aging populations where oxidative stress and chronic inflammation are prominent. Given the increasing burden of aging-related COPD and the emerging biological effects of molecular hydrogen, we hypothesized that H_2_ therapy may exert beneficial effects by modulating oxidative stress, inflammation, and aging pathways.

**Methods:**

Following Ferrari’s narrative review framework, PubMed and Ovid MEDLINE were searched through October 2025 for clinical and preclinical studies examining H_2_ interventions in COPD, chronic bronchitis, or emphysema, with emphasis on elderly populations or aging-related models.

**Results:**

Three studies met the inclusion criteria: one animal experiment, one prospective clinical study, and one randomized controlled trial. H_2_ administration, via inhalation or H_2_-rich water, significantly reduced inflammatory cytokines (IL-1β, IL-6, TNF-α), oxidative markers (8-OHdG), and senescence indicators (p16, p21, β-gal). Clinical findings demonstrated improved arterial blood gases, acid–base balance, and exercise tolerance in elderly COPD patients.

**Conclusions:**

H_2_ therapy shows consistent antioxidative, anti-inflammatory, and anti-senescence effects across preclinical and clinical settings, suggesting potential as an adjunctive treatment for COPD in aging populations. Further large-scale, long-term trials are warranted to confirm efficacy, optimize dosage, and clarify mechanistic pathways.

## What is known?

Molecular hydrogen (H_2_) has demonstrated antioxidative and anti-inflammatory properties across various diseases, including respiratory disorders such as COPD. However, its specific therapeutic relevance to aging-related COPD pathophysiology remains insufficiently clarified.

## What new information does this article contribute?

This narrative review integrates preclinical and clinical evidence showing that H_2_ therapy attenuates oxidative stress, inflammation, and cellular senescence in both aging models and elderly COPD patients – highlighting its potential as a novel adjunctive intervention targeting aging mechanisms in chronic respiratory care.

## Introduction

Molecular hydrogen (H_2_), owing to its unique antioxidant, anti-inflammatory, and anti-apoptotic properties, has emerged as a highly promising therapeutic agent in modern medicine [1]. Since its discovery by Antoine Lavoisier in 1783, H_2_ was initially regarded as an inert gas without biological activity [1]. However, this perception has gradually shifted, and in the 21st century, the therapeutic potential of molecular hydrogen has gained increasing recognition [2], [3], [4], [5], [6], [7]. H_2_ possesses several advantages, including a small molecular size, colorless and odorless characteristics, rapid tissue diffusion, and negligible toxicity even at therapeutic concentrations [6], 8]. H_2_ exerts its antioxidant effect by selectively reducing highly reactive molecules, including hydroxyl radicals (•OH) and peroxynitrite (ONOO^−^), while sparing other physiologically essential reactive oxygen species (ROS) [1], 6]. This selective action helps to minimize potential adverse effects on normal cellular signaling. H_2_ therapy has demonstrated promising therapeutic effects across a wide range of conditions, including inflammatory diseases, cancers, and respiratory disorders [9], [10], [11], [12], [13], [14]. H_2_ therapy can be administered via multiple routes, including inhalation, oral hydrogen capsules, hydrogen baths, hydrogen-rich saline injections, and topical eye drops [15].

The primary mechanisms of H_2_ therapy include antioxidant mechanisms, such as selective radical scavenging, mitochondrial protection, and activation of the nuclear factor erythroid 2-related factor 2 (Nrf2) pathway [6], [16], [17], [18], [19]; anti-inflammatory effects, including inhibition of the NLR family pyrin domain containing 3 (NLRP3) inflammasome and nuclear factor kappa-light-chain-enhancer of activated B cells (NF-κB), as well as immunomodulation through macrophage polarization [15], 20], 21]; and anti-apoptotic and cytoprotective effects [22], [23], [24].

Recent reviews have further explored the protective effects of H_2_ in pulmonary diseases, including chronic obstructive pulmonary disease (COPD), asthma, pulmonary fibrosis, pulmonary hypertension, acute lung injury, lung cancer, and COVID-19 [1]. Among these, COPD stands out as a particularly relevant area of investigation in the context of aging. COPD represents a major chronic respiratory disease characterized by persistent symptoms and irreversible airflow limitation [25], 26]. Aging is a major risk factor for the development and progression of COPD, which imposes a growing health burden on the elderly population due to its increasing prevalence and complex management challenges [25], 27]. Therefore, given the increasing burden of aging-related COPD and the emerging biological effects of molecular hydrogen, we hypothesized that H_2_ therapy may exert beneficial effects on COPD by modulating oxidative stress, inflammation, and aging-related pathways. Accordingly, this narrative review aimed to answer the following research question: What evidence exists regarding the therapeutic and mechanistic roles of H_2_ therapy in COPD in the context of aging?

## Methods

This narrative review was conducted using the methodological principles proposed by Ferrari’s narrative review framework, which emphasizes a transparent, structured, and reproducible approach to literature identification, selection, extraction, and synthesis [28]. The review was designed to provide an integrative overview of the therapeutic effects and physiological mechanisms of H_2_ in COPD, chronic bronchitis, and emphysema, with particular attention to aging-related mechanisms and elderly populations. Although this review was not conducted as a systematic review, key elements of structured literature searching, predefined eligibility criteria, independent screening, and standardized data extraction were incorporated to enhance methodological rigor. As this study was based solely on previously published literature that Ethical Approval was not required.

### Eligibility criteria

We included peer-reviewed studies that investigated the therapeutic effects or physiological mechanisms of H_2_ in patients with COPD, chronic bronchitis, or emphysema. Studies focusing on elderly or aging populations, or those examining aging-related factors, were prioritized. Both clinical and preclinical studies were considered eligible if they examined outcomes related to oxidative stress, inflammation, respiratory function, or quality of life. No language restrictions were applied.

### Information sources

Following Ferrari’s narrative review framework, a structured literature search was conducted to identify relevant studies [28]. PubMed and Ovid MEDLINE were searched from database inception to October 22, 2025. In addition, reference lists of eligible studies and relevant reviews were manually screened to identify additional articles that could provide important contextual or mechanistic evidence.

### Search strategy

The search strategy combined controlled vocabulary terms (e.g., Pulmonary Disease, Chronic Obstructive [Mesh], Aged [Mesh]) with free-text keywords related to molecular hydrogen therapy. Keywords included “molecular hydrogen,” “hydrogen gas,” “hydrogen-rich water,” “hydrogen therapy,” “hydrogen inhalation,” “chronic obstructive pulmonary disease,” “COPD,” “chronic bronchitis,” “emphysema,” “obstructive lung disease,” “elderly,” “older adults,” and “geriatric.” Search strategies were adapted for each database (PubMed and Ovid MEDLINE) to ensure both sensitivity and specificity.

### Study selection and data extraction

Two reviewers (Y-T. C and T-A. C) independently screened the titles and abstracts of all retrieved records after the database search. Articles deemed potentially eligible were retrieved for full-text review. Any disagreements during the selection process were resolved through discussion, and a third reviewer (C. T) was consulted when necessary. When essential information was missing or unclear, attempts were made to contact the original study authors.

A standardized data extraction form was developed to collect the following information from each included study: (1) bibliographic details (title, authors, year of publication, and journal); (2) study characteristics (design, country/region, study period, and intervention/comparator details); (3) participant characteristics; and (4) reported outcomes. Data extraction was performed independently by two reviewers (Y-T. C and T-A. C) to ensure accuracy and consistency.

## Results

### Search results

The database search process and study selection were conducted according to Ferrari’s narrative review framework [28]. A total of 3 studies met the inclusion criteria after screening 3 records retrieved from PubMed (n=2) and Ovid MEDLINE (n=2). One duplicate record was excluded before screening. No additional reports were identified from other sources. All three reports were assessed for eligibility and included in the final review.

### Study selection and characteristics of included studies

Three studies were included, spanning China (n=1), Japan (n=1), and Russia (n=1). Study designs included one preclinical animal experiment [29], one prospective clinical study [30], and one randomized controlled clinical trial [31]. Together, these studies explored the therapeutic and mechanistic roles of H_2_ therapy in COPD or aging-related factors (Table 1).

**Table 1:** Characteristics of included studies on molecular hydrogen therapy in COPD and aging-related conditions.

First Author (Year)	Country	Study design/model	Participants	Setting	Intervention/administration	Outcomes measured
Wang S-T et al. [30]	China	Prospective clinical study	20 patients (10 COPD, 10 asthmas; COPD group age 52–70 y)	Tertiary hospital outpatient	Inhalation of 2.4 % H_2_ steam-mixed gas (“XEN”) for 45 min once	Granulocyte-macrophage colony stimulating factor, interferon-γ, IL-1β, IL-2, IL-4, IL-5, IL-6, IL-8, IL-10, IL-13, IL-17A, MIP-1α, MIP-1β, TNF-α Differentiation-40 ligand, MCP-1, vascular endothelial growth factor A SOD3
Suzuki Y et al. [29]	Japan	Preclinical animal experiment (SMP30-KO aging-lung model)	48 male mice (aging-relative gluconolactonase: SMP30-KO)	Controlled laboratory	Drinking H_2_-rich pure water (∼7 ppm) twice daily	Histology: MLI, DI Lung mechanics Immunohistochemistry: oxidative DNA damage (γH2AX, 8-OHdG) Senescence markers (p16, p21, β-gal)
Shogenova LV [31]	Russia	Randomized controlled clinical trial	100 COPD patients (GOLD C–D; age 66–78 y)	Hospital ICU	Daily × 14 days with O_2_ + NIV support: Group 1 (n=22) t-He/O_2_ → NO → H_2_ Group 2 (n=20) t-He/O_2_ + NO Group 3 (n=20) t-He/O_2_ + H_2_ Group 4 (n=18) NO + H_2_ Group 5 (n=20) control with O_2_ only.	Blood gas (PaO_2_, PaCO_2_), acid–base (HCO_3_^−^, lactate), hemodynamics, 6-min walk distance

8-OHdG, 8-hydroxy-2′-deoxyguanosine; COPD, chronic obstruction pulmonary disease; DI, destructive index; EBC, exhaled breath condensate; ICU, intensive care unit; IL, interleukin; MCP-1, monocyte chemotactic protein 1; MIP-1α, macrophage inflammatory protein 1α; MLI, mean linear intercept; NIV, non-invasive ventilation; SOD3, superoxide dismutase 3; SMP30-KO, senescence marker protein 30 knockout; TNF-α, tumor necrosis factor alpha; ɤH2AX, phosphorylated histone H2AX; y, years old.

An experimental study administered H_2_-rich water (∼7 ppm) twice daily in an senescence marker protein 30 knockout (SMP30-KO) murine model to mimic aging-related pulmonary decline [29]. Intervention protocols varied across settings. In one outpatient study, participants received a single 45-min session of 2.4 % H_2_ steam-mixed gas inhalation [30]. In a recent ICU trial, patients with advanced COPD underwent daily H_2_ inhalation for 14 days, with five treatment subgroups combining H_2_, NO, and He/O_2_ gas mixtures under non-invasive ventilation support [31].

### Participants and study settings

Across studies, total sample sizes ranged from 20 patients in clinical outpatients to 100 ICU patients in the randomized trial and 48 SMP30-KO mice in the aging model [29], [30], [31]. Participant ages in human studies ranged between 52 and 78 years, aligning with aging and COPD-relevant populations [30], 31]. Clinical investigations were conducted in tertiary hospital and ICU settings, while preclinical work was performed under controlled laboratory conditions [29], [30], [31].

### Outcomes and mechanistic markers

Measured outcomes collectively encompassed inflammation, oxidative stress, cellular senescence, and respiratory function. The preclinical study revealed that H_2_ administration modulated inflammatory cytokines, including interleukin-1 beta (IL-1β), interleukin-6 (IL-6), and tumor necrosis factor-alpha (TNF-α); chemokines such as monocyte chemoattractant protein-1 (MCP-1) and macrophage inflammatory protein-1 alpha/beta (MIP-1α/β); and the antioxidant enzyme extracellular superoxide dismutase (SOD3) [30]. Another investigation demonstrated that H_2_-rich water attenuated oxidative DNA damage, indicated by reduced phosphorylated histone H2AX (γH2AX) and decreased 8-hydroxy-2′-deoxyguanosine (8-OHdG) levels, as well as lowered expression of senescence markers (p16, p21, β-gal) in the aging-lung model [29]. In the randomized controlled clinical trial, H_2_ inhalation improved arterial blood gases, acid–base balance, and 6-min walk distance, accompanied by decreased 8-OHdG levels, indicating enhanced oxidative resilience and functional recovery [31].

### Methodological quality and risk of bias

The clinical studies exhibited moderate methodological rigor, though limitations included small sample sizes and incomplete blinding [30], 31]. The animal model provided strong mechanistic evidence but limited direct clinical generalizability [29]. Overall, the included evidence supports the antioxidative, anti-inflammatory, and anti-senescence effects of H_2_ within both aging-related and COPD contexts.

## Discussion

In the present study, we found that H_2_ therapy demonstrates consistent antioxidative, anti-inflammatory, and anti-senescence effects across both preclinical and clinical studies of COPD, particularly within aging-related contexts. COPD is a complex and heterogeneous respiratory disorder with high morbidity and mortality, causing more than three million deaths annually worldwide and imposing a substantial burden on global healthcare and financial systems [32], 33]. Traditionally, the major risk factors have been attributed to long-term exposure to cigarette smoke, air pollution, and genetic susceptibility; however, the aging process also plays a crucial role in the pathogenesis of COPD [34], [35], [36]. Aging represents a progressive physiological and pathophysiological decline in organismal function over time, involving inflammation, oxidative stress, mitochondrial dysfunction, epigenetic alterations, cellular senescence, and apoptosis [25], 37]. Studies have shown that the prevalence of COPD increases with age, with individuals aged ≥65 years having a fivefold higher risk compared with those under 40 years [36], 38].

Recent bioinformatics analyses have identified 24 genes associated with both aging and COPD, mainly participating in the TNF, NF-κB, IL-17, and HIF-1 signaling pathways, all of which are implicated in the pathophysiological mechanisms of COPD [25], 39], 40]. The study further pinpointed four key aging- and COPD-related genes (HIF1A, CDKN1A, MXD1, and SOD2) that may directly influence COPD progression and revealed that hsa-miR-519d-3p may also be involved in the aging–COPD interaction [25], [41], [42], [43]. Although the precise relationship between aging and COPD remains incompletely understood, substantial evidence supports their close association [25], 27], 36], 38].

**Figure 1:**
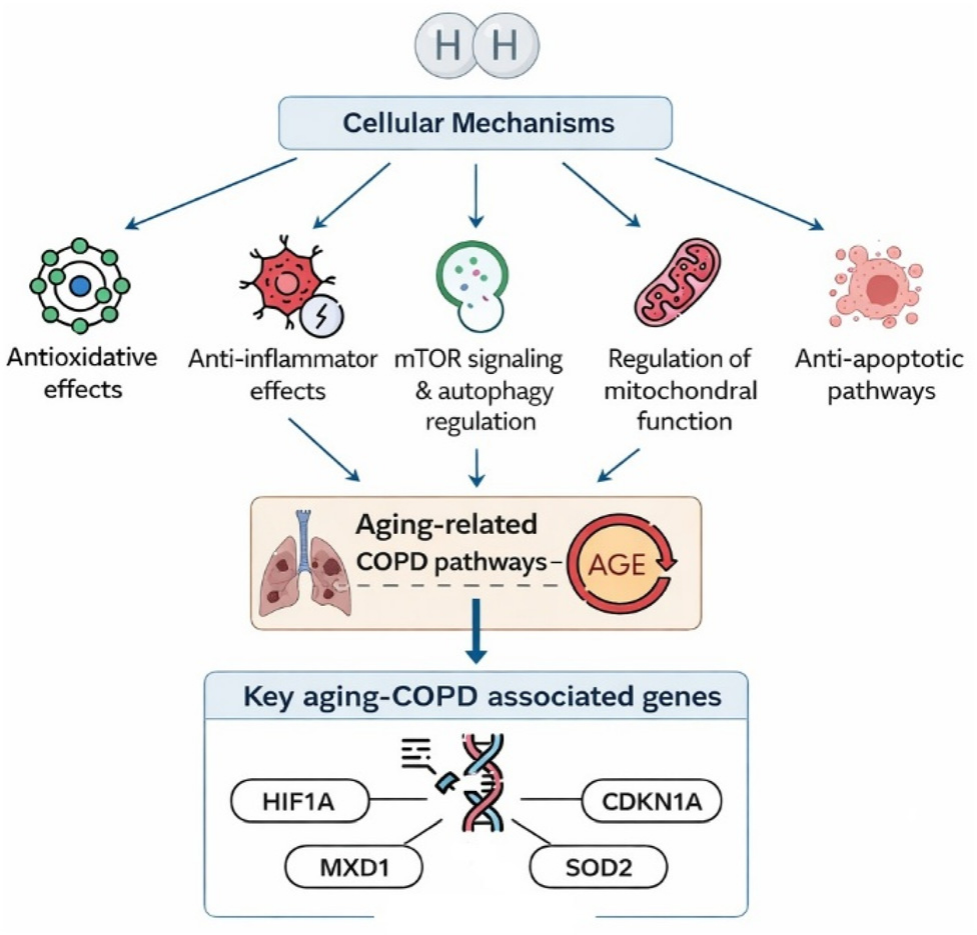
Proposed mechanisms of molecular hydrogen (H_2_) in COPD and aging.

H_2_ therapy, as an emerging therapeutic approach, antioxidant, anti-inflammatory, mTOR and autophagy-modulating, mitochondrial-regulatory, and anti-apoptotic effects [37]. These mechanisms are of great relevance to both anti-aging and COPD management [44], [45], [46], [47]. Moreover, due to its rapid and convenient diffusion into lung tissue and its direct action on pulmonary cells, hydrogen therapy has recently shown promising therapeutic potential in COPD [44], 48]. These proposed mechanisms are illustrated in Figure 1. It is these intersections that led us to initiate this study. The findings across the included studies collectively highlight H_2_ as a promising therapeutic adjunct for COPD and aging-related respiratory dysfunction. Although these investigations differed in design and population – from animal models to elderly clinical cohorts – they consistently demonstrated that H_2_ exerts antioxidative, anti-inflammatory, and cytoprotective effects relevant to both COPD pathophysiology and aging mechanisms [29], [30], [31].

In a preclinical study, the SMP30-KO model provided key mechanistic evidence linking H_2_ therapy to the mitigation of aging-associated lung vulnerability [29]. H_2_-rich saline attenuated cigarette smoke–induced emphysema, restored lung compliance, and reduced oxidative DNA damage (8-OHdG) as well as cellular senescence markers (p16/p21), indicating that H_2_ directly targets oxidative and senescence pathways implicated in age-related pulmonary decline [29], [49], [50], [51], [52]. These findings established a biological foundation for translation into elderly COPD populations. Building upon these mechanistic insights, a prospective clinical study evaluated short-term H_2_ inhalation in patients with COPD and asthma [30]. The intervention significantly decreased inflammatory cytokines such as IL-6 and MCP-1 and increased soluble CD40 ligand levels in exhaled breath condensate, suggesting that H_2_ modulates redox balance and immune signaling in the human airway [30], [53], [54], [55]. Although the study population consisted mainly of middle-to-older adults, the anti-inflammatory and antioxidative trends observed support future application in elderly cohorts, where chronic inflammation and oxidative stress are more pronounced [30].

Further clinical validation was provided by a randomized controlled trial, which demonstrated that combined gas therapy incorporating H_2_, nitric oxide (NO), and helium/oxygen (He/O_2_) improved arterial blood gases, reduced hypercapnia and vascular stiffness, and enhanced exercise tolerance in elderly patients (≥65 years) [31]. The observed decreases in inflammatory markers and improvements in endothelial function indicate that H_2_ contributes synergistically within multimodal respiratory rehabilitation, particularly in post-COVID and geriatric COPD populations [31].

This narrative review has several limitations. First, the number of included studies was limited and the study designs were heterogeneous, including a preclinical animal experiment, a pilot clinical study, and a randomized clinical trial. The preclinical study may have limited direct clinical generalizability, and variability in hydrogen intake may have occurred due to free access to hydrogen-rich water and rapid decline in hydrogen concentration. Second, the clinical evidence was based on relatively small sample sizes and short intervention durations, with uncertainties regarding optimal dosing and treatment protocols. Finally, one randomized trial evaluated a combined gas therapy (thermal heliox, nitric oxide, and hydrogen), making it difficult to isolate the independent effect of molecular hydrogen, and the study population consisted primarily of post-COVID COPD patients, which may limit broader generalizability.

Despite these limitations, the available evidence suggests that H_2_ therapy may mitigate inflammation and oxidative stress while potentially improving respiratory mechanics and aging-related pulmonary conditions. Although the short-term safety of H_2_ inhalation therapy has been repeatedly reported, further large-scale and long-term studies are required to confirm sustained clinical benefits, determine optimal dosing strategies, and clarify how H_2_ therapy can be integrated with standard COPD management and geriatric rehabilitation programs.

## Conclusions

This narrative review highlights H_2_ as a promising adjunctive therapy for COPD, particularly in aging-related contexts. Across preclinical and clinical investigations, H_2_ demonstrated consistent antioxidative, anti-inflammatory, and anti-senescence effects that collectively contribute to improved respiratory function and oxidative resilience. The SMP30-KO murine model established mechanistic links between H_2_ and the attenuation of aging-related pulmonary vulnerability, while clinical studies in elderly patients revealed improvements in arterial blood gases, exercise tolerance, and oxidative stress markers. These findings suggest that H_2_ therapy may target fundamental aging mechanisms – such as mitochondrial dysfunction, redox imbalance, and chronic inflammation – that underlie COPD progression. Nevertheless, the current evidence base remains limited by small sample sizes and short intervention durations. Future large-scale, longitudinal, and mechanistic studies are warranted to confirm efficacy, define optimal administration routes and dosages, and explore integration with conventional COPD management and geriatric rehabilitation strategies.
